# Sedimentary and geochemical characteristics of two small permafrost-dominated Arctic river deltas in northern Alaska

**DOI:** 10.1007/s41063-018-0056-9

**Published:** 2018-06-22

**Authors:** Matthias Fuchs, Guido Grosse, Benjamin M. Jones, Jens Strauss, Carson A. Baughman, Donald A. Walker

**Affiliations:** 1 Alfred Wegener Institute, Helmholtz Centre for Polar and Marine Research, Telegrafenberg A45, 14473 Potsdam, Germany; 2 Institute of Earth and Environmental Sciences, University of Potsdam, Karl-Liebknecht-Str. 24-25, 14467 Potsdam, Germany; 3 Water and Environmental Research Center, University of Alaska Fairbanks, 437 Duckering, PO Box 755860, Fairbanks, AK 99775 USA; 4 Alaska Science Center, U.S. Geological Survey, 4210 University Drive, Anchorage, AK 99508 USA; 5 Alaska Geobotany Center, Institute of Arctic Biology, University of Alaska Fairbanks, 311 Irving, PO Box 757000, Fairbanks, AK 99775 USA

**Keywords:** Alaska North Slope, Soil organic carbon, Accumulation rates, Radiocarbon dating, Upscaling, Landsat 8, Supervised classification

## Abstract

Arctic river deltas are highly dynamic environments in the northern circumpolar permafrost region that are affected by fluvial, coastal, and permafrost-thaw processes. They are characterized by thick sediment deposits containing large but poorly constrained amounts of frozen organic carbon and nitrogen. This study presents new data on soil organic carbon and nitrogen storage as well as accumulation rates from the Ikpikpuk and Fish Creek river deltas, two small, permafrost-dominated Arctic river deltas on the Arctic Coastal Plain of northern Alaska. A soil organic carbon storage of 42.4 ± 1.6 and 37.9 ± 3.5 kg C m^− 2^ and soil nitrogen storage of 2.1 ± 0.1 and 2.0 ± 0.2 kg N m^− 2^ was found for the first 2 m of soil for the Ikpikpuk and Fish Creek river delta, respectively. While the upper meter of soil contains 3.57 Tg C, substantial amounts of carbon (3.09 Tg C or 46%) are also stored within the second meter of soil (100–200 cm) in the two deltas. An increasing and inhomogeneous distribution of C with depth is indicative of the dominance of deltaic depositional rather than soil forming processes for soil organic carbon storage. Largely, mid- to late Holocene radiocarbon dates in our cores suggest different carbon accumulation rates for the two deltas for the last 2000 years. Rates up to 28 g C m^− 2^ year^− 1^ for the Ikpikpuk river delta are about twice as high as for the Fish Creek river delta. With this study, we highlight the importance of including these highly dynamic permafrost environments in future permafrost carbon estimations.

**Electronic supplementary material:**

The online version of this article (10.1007/s41063-018-0056-9) contains supplementary material, which is available to authorized users.

## Introduction

Arctic river deltas are dynamic environments at the interface between land and sea [1]. They are characterized by typical fluvial and deltaic processes such as tides, periodic flooding, sediment deposition, channel migration, and shoreline erosion. Compared to low-latitude river deltas, harsh Arctic winters lead to pronounced seasonal runoff patterns with low or no discharge in winter, a peak discharge during the time of river ice break-up, and a declining discharge until freeze-up. A unique aspect of Arctic river deltas is that large parts of the landscape are underlain by permafrost. The presence of permafrost results in different surface features (e.g., patterned ground, ice wedge polygons, frost mounds) as well as sediment erosion dynamics [2, 3]. Due to the often high ground ice content in fine-grained permafrost deposits, Arctic river deltas are prone to thermokarst and thermo-erosion processes [4]. Thaw-induced surface settling, lake formation, active layer thickening, enhanced thawing of the sub-aquatic permafrost under lakes and delta channels, as well as lake drainage and permafrost aggradation, shore erosion and channel migration, lead to an overall heterogeneous and dynamic landscape in Arctic river deltas [5].

These dynamic environments are important when assessing carbon (C) and nitrogen (N) feedbacks from permafrost soils. In general, permafrost deposits contain significant amounts of frozen organic carbon (e.g., Tarnocai et al. [6], Hugelius et al. [7]) vulnerable to thaw and mobilization in a changing climate [8]. While several investigations assess the C storage potential of permafrost soils in the Arctic [6, 9–18], only a few studies have included N stock estimations [19–24]. Whereas organic C in permafrost soils has the potential to increase atmospheric CO_2_ and CH_4_ concentration [8, 25–28], N is often a limiting factor for plant growth in tundra environments [29–33]. Therefore, it is important to assess N as well as C storage in permafrost landscapes for inventorying and future modeling efforts.

Permafrost C and N storage in soils of Arctic river deltas is both complex and poorly constrained. Only a few studies have focused on Arctic river deltas specifically and limited field data is available on C or N stocks in Arctic deltas, e.g., Ping et al. [19] have included data from 13 sites located in Arctic river deltas in their study on C and N pools along the Alaskan Beaufort Sea Coast of Alaska. Zubrzycki et al. [22] estimated organic C and N pools for different sites within the Lena river delta and Shmelev et al. [34] included deltaic deposits in their field data compilation from North-Eastern Yakutia. Tarnocai et al. [6] included five soil profiles from the Mackenzie Delta to infer a first-order estimate of 241 Pg C for Arctic deltaic deposits below 3 m depth for their circum-polar permafrost soil carbon estimation. The most recent permafrost soil C stock estimation includes 12 major Arctic river deltas^1^ and estimated their total soil organic carbon (SOC) pool to be 91 ± 52 Pg C for deposits below 3 m [7]. However, only data from the two largest deltas Lena and Mackenzie as well as the data from the study of Ping et al. [19] were included and extrapolated to represent a land area for deltas of 75,800 km^2^. All other Arctic river deltas, in particular the large number of small- and medium-sized deltas, remain unaccounted for in permafrost deltaic C stock assessments [7]. Thus, C and N stocks in Arctic river deltas remain highly uncertain.

This study presents soil organic carbon, nitrogen and radiocarbon results from two small (~ 100 km^2^) Arctic river deltas located on the Arctic Coastal Plain (ACP) of northern Alaska—the Ikpikpuk River (IKP) and the Fish Creek River (FCR) deltas. Soil permafrost cores were collected and analyzed with the aim (1) to estimate SOC and N stocks for the FCR and IKP deltas down to 2 m depth and scaling it up to the landscape level based on Landsat satellite imagery, and (2) to analyze C accumulation rates for a better understanding of permafrost C dynamics in Arctic river deltas.

## Study area

**Fig. 1:**
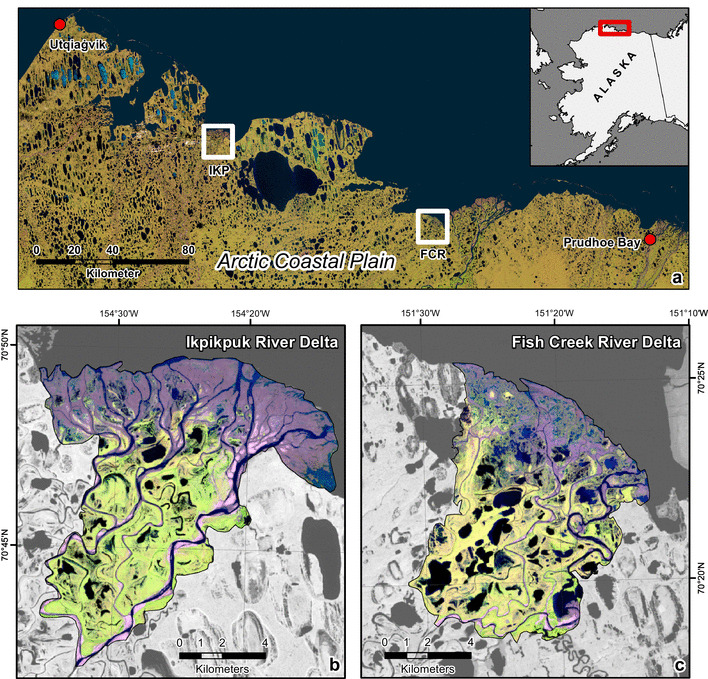
**a** The central Arctic Coastal Plain of northern Alaska (Landsat 8 satellite image mosaic) including the location of the two study areas Ikpikpuk (IKP) and the Fish Creek (FCR) river delta between Utqiaġvik and Prudhoe Bay. **b** The triangle-shaped IKP river delta with its sandy fan (purple) in the forefront of the delta in comparison to **c** the rectangular-shaped FCR river delta without a distinct sandy fan. Both **b** and **c** are Landsat 8 false color composites (bands 6–5–4, acquisition date: 5 August 2016)

The IKP and FCR river deltas are located on the ACP of northern Alaska (Fig. 1) and, like their entire catchments, are part of the continuous permafrost zone [35]. According to surficial geology maps [36, 37], both river deltas are dominated by alluvium deposits consisting of fine to medium sand and silty sand. Both river deltas are similar in size, the IKP delta has an aerial extent of 106 km^2^ and the FCR delta covers 87 km^2^. We classify these deltas, therefore, as small Arctic river deltas compared to the area of the nine largest Arctic deltas of the Lena (32,000 km^2^), Mackenzie (13,000 km^2^), Yana (6600 km^2^), Indigirka (5000 km^2^) Yenisei (4500 km^2^), Kolyma (3200 km^2^) Ob (3200 km^2^), Pechora (3200 km^2^) and Yukon (3000 km^2^) rivers [1].

The IKP delta is fan-shaped and located 100 km southeast of Utqiaġvik (Barrow) Alaska. It is dominated by coastal wet sedge tundra [38] and thermokarst processes have affected 37% of the IKP delta during the Holocene [4]. The Ikpikpuk River is a meandering stream on the Alaska North Slope with a low height gradient. We determined the IKP watershed to cover 15,330 km^2^ and the highest point in the catchment lies at about 468 m a. s. l.

The FCR delta has a more rectangular shape and is located approximately 110 km west of Prudhoe Bay, Alaska, in close vicinity to the well-studied Colville river delta [2, 5, 39–42]. The FCR delta consists of the same vegetation type as the IKP delta; however, most of the delta forefront consists of coastal marsh instead of barren (sandy) deposits [43]. It drains a watershed of 4815 km^2^, an area about one-third of the Ikpikpuk watershed, with the highest point located at 336 m a. s. l. Both rivers drain large portions of the Ikpikpuk Sand Sea, which consists of stabilized Pleistocene dune fields affected by thermokarst processes [44, 45].

The mean river discharge at the Ikpikpuk gauge station (NWIS ID#15820000, 69°46′00.5″N; 154°39′40.6″W, NAD27) is approximately 25.5 m^3^ s^− 1^ (2005–2009). Discharge at the Fish Creek gauge station (NWIS ID#15860000, 70°16′14″N; 151°52′09″W, NAD27), is approximately 5.4 m^3^ s^− 1^ (2005–2009). The peak flow occurs from late May to early June in both rivers [46, 47]. Most of the sediment load occurs presumably during the river ice break-up and the subsequent spring flood, similar to the adjacent Colville river delta [39, 48]. The groundwater contribution to the rivers discharge likely is small due to the continuous permafrost in the watersheds [42]. Mean annual air temperature is − 10.2 °C at the Ikpikpuk climate station (2006–2010) and − 9.9 °C (2006–2010) at the Fish Creek climate station [49], with a mean summer precipitation of < 200 mm for the Fish Creek climate station [47].

## Materials and methods

### Soil organic carbon and soil nitrogen storage

Soil samples were collected in April 2014 in the FCR delta and in July 2015 in the IKP delta. In total, nine cores were collected. Core lengths vary from 54 to 215 cm. Core characteristics and sample locations including a map are presented in the Supplementary Material. For the July 2015 cores, prior to drilling the permafrost soil cores with a Snow, Ice and Permafrost Research Establishment (SIPRE) auger barrel drill (Jon’s Machine Shop, Fairbanks, USA), the active layer was excavated and sampled with fixed volume cylinders. Therefore, the entire soil profile from the surface to a depth of 2 m was sampled where possible. Cores collected in April 2014 were transported frozen and intact to the laboratory where they were subsampled. Cores collected in July 2015 were subsampled in ~ 5 cm long pieces in the field considering the core cryostratigraphy and core sediment characteristics. The samples were transported cool to the laboratory facilities at Alfred Wegener Institute (AWI) Potsdam. All soil cores were photographed, described for cryostratigraphy, lithology and plant macrofossils.

In the laboratory, 80 samples from the IKP delta and 49 samples from the FCR delta were analyzed for geochemical parameters. All samples were freeze-dried, homogenized, ground with an agate-ball mill, and then analyzed with a Vario EL III Elemental Analyzer for total carbon (TC) and total nitrogen (TN). In a next step, total organic carbon (TOC) was measured with the Vario Max C Elemental Analyzer. As a result, the carbon–nitrogen ratio (quotient of TOC and TN) as well as the amount of total inorganic carbon (difference between TC and TOC) were calculated. In addition, the dry bulk density was determined by dividing the dry weight of a sample by its original volume and expressed in g cm^− 3^, and the volumetric ice content was calculated as quotient of the volume of ice within a particular sample and the sample volume itself.

Permafrost soil organic carbon (SOC) and soil nitrogen (SN) storage were calculated for each sample by multiplying the dry bulk density, the percentage organic carbon or nitrogen, the mineral fraction (< 2 mm) and the sample length according to Michaelson et al. [9]. SOC and SN storages for the samples were summed up for the reference depths of 30, 100, and 200 cm based on the field notes, which allowed a weighted summation considering the core lithology. Likewise, missing core intervals were filled by extrapolation from adjacent samples of the same core depending on the lithology and cryostratigraphy according to the field notes. When a permafrost core was shorter than 200 cm, SOC and SN were extrapolated to the next reference depth, based on the lowest sample; however, extrapolation never exceeded 50 cm below the lowest sample.

### Radiocarbon dating and organic carbon accumulation rates

A subset of 24 samples was wet-sieved with a 2-µm sieve and plant macro remains were handpicked under a microscope and submitted for age determination to the radiocarbon laboratory in Poznan, Poland. The samples were analyzed with the accelerated mass spectrometer (AMS) approach [50]. The resulting radiocarbon ages were calibrated with Calib 7.1 software into calibrated years before present (cal yr BP) [51, 52]. Post-bomb radiocarbon dates were converted into F^14^C (fraction modern) and calibrated with the ^14^Chrono, intcal13 NHZ1 post-bomb calibration data set [53, 54]. For the analysis and comparison between post-bomb radiocarbon dates and older dates, all calibrated data were converted into years before the year 2000 (cal yr B2000).

Based on the radiocarbon ages, sediment accumulation rates as well as SOC accumulation rates were calculated. SOC accumulation rates are based on the cumulative SOC at the depth of the radiocarbon sample. Therefore, SOC was summed to the depth of the radiocarbon sample and then divided by the calibrated years. Sediment accumulation rates were calculated similarly. The sediment height from the depth of the radiocarbon dated sample to the surface was divided by the calibrated year of the corresponding sample. In addition, intra-sedimentary ice content was accounted for in the calculations to avoid overestimation of sediment accumulation rates.

### Grain-size distribution

Grain-size is an important indicator of the conditions under which the sediment was deposited. High-energy main channel deposits may accumulate organic matter differently than low-energy backwater deposits. For our study, 104 samples were analyzed for grain-size distribution. To remove organic components prior to measuring, 10 ml of hydrogen peroxide (30%) was added to the sample three times a week for 5 weeks. The organic-free samples were washed to achieve a neutral pH by centrifugalizing. A 1% ammonia solution and 1 g of dispersing agent (Na_4_P_2_O_7_ × 10 H_2_O) were added to disperse the samples on an overhead shaker for 12 h. Samples were then equally split in sub-samples whereof at least three sub-samples were measured with the Malvern Mastersizer 3000. The samples were measured in the wet dispersion unit with deionized water as dispersant and each measurement process included three repeated measurements. As a result, at least nine measurements were averaged for each sample and the result is displayed in volume percentage. Results were analyzed with GRADISTATv8 [55] to determine the sorting and sample distribution, and using the soil texture package [56] in the open source software R (version 3.5.0) [57]. To generate soil texture triangles and classify the samples according to the USDA soil texture system [58], results were converted into weight percentages (wt%) using particle density values by Schjønning et al. [59].

### Scaling soil core carbon and nitrogen stocks to landscape level

SOC and SN stocks were scaled up from the soil core storages to the entire delta using two different methods. The first method used mean core SOC and SN values of all the soil cores for each respective delta. The mean vales were scaled up based on the spatial extent of the river delta area. This standard ‘averaging-approach’ does not take into consideration any landscape differences but allows a first estimation of the potential SOC and SN stored in the two study areas.

The second method used a more detailed scaling up of C and N stocks by including a remotely sensed land cover classification (LCC). This allows a more refined and weighted scaling of C and N stocks based on surface coverage, even though there were only two cores per land cover class. We are aware that this data basis is statistically very limited due to the low number of sampled soil cores and as such the landscape-scale SOC and SN stocks from this upscaling should be seen as a first-order estimation. The upscaling is based on a 30 m resolution Landsat 8 image. The cloud free image, acquired on 5 August 2016 (Source: U.S. Geological Survey, Earth Explorer) covered both deltas and allowed a supervised classification including training areas from both river deltas. A maximum likelihood classification was carried out in ArcGIS 10.4 to distinguish the six major landscape units in the deltas. The final LCC included the following classes: ‘Water’, ‘Barren land’, ‘Sparsely vegetated’, ‘Moist sedge tundra’, ‘Wet sedge tundra’, and ‘Coastal marsh’. A similar classification scheme was applied in a land cover study in the Lena Delta by Schneider et al. [60]. Since there were no permafrost soil cores collected in the class ‘Coastal marsh’, this class was merged for the upscaling with the class ‘Wet sedge tundra’. For the scaling up of the C and N stocks, soil permafrost cores from both Arctic river deltas were combined and mean values for the different land cover classes were calculated. Based on the mean values of collected soil cores within a class and the areal coverage of a particular land cover class, the total SOC and SN were then calculated. Water areas were excluded from the analysis.

In all the SOC and SN landscape stock calculations, landscape ice wedge volume was accounted for to avoid overestimation. While ice wedges do contain dissolved organic carbon (DOC), this pool likely is very small compared to the particulate carbon in the sediments [61]. Due to a lack of DOC data in ground ice and soils of our study sites, we, therefore, did not consider this pool and ice wedges were assumed to contain no C or N. For both study areas, a landscape-scale ice wedge content of 6% was included derived from the study of Kanevskiy et al. [62], who investigated ground ice characteristics in 12 different study sites in deltas and tidal flats along the Beaufort Sea Coast of Alaska.

The validation of the LCC was based on a high resolution (2.5 m), false color, infrared, aerial-image ortho-photos (ID: DI00000100016777 and ID: DI00000100203093, acquisition date: 18 July 2002, U.S. Geological Survey, DOQ, Earth Explorer) in combination with photographs taken from overflights in summer 2015. We manually classified 63 points, not overlapping with the training areas, according to the orthophoto and the aerial photos and cross-checked it with the LCC to determine the overall accuracy and the kappa index of agreement.

## Results

### Carbon and nitrogen contents

The TOC contents for the IKP delta samples range from 0.3 (sand) to 24.5% (peat), with a median value for all the samples of the IKP delta of 3.0% (− 2.0/+ 2.2; 25th and 75th percentiles). The FCR delta had a higher median value due to fewer samples collected in sandy deposits with a median of 7.6% (− 3.2/+ 4.4) and values ranged from 0.7% (silty sand) to a maximum value of 30.3% (peat). Sample N contents were in most cases below 1% with a median value for the IKP delta samples of 0.2% (− 0.1/+ 0.1) and for the FCR delta samples of 0.5% (− 0.2/+ 0.2). Many sandy samples from the IKP delta had a N content below 0.1%, the detection limit of the elemental analyzer.

In addition, based on sample TOC and TN contents, soil core SOC and SN stocks were calculated for the different reference depths. Generally, lower SOC and SN stocks in the first meter of soil were found in the IKP delta compared to the FCR delta. SOC and SN stocks of the reference depths for all soil cores are provided in Table 1. Soil core SOC stocks ranged from 16.5 kg C m^− 2^ (0–100 cm) for a site on barren land (sand bank) in the IKP delta to 32.4 kg C m^− 2^ (0–100 cm) for a wet tundra site in the FCR delta. Mean soil core SOC stocks (± standard deviation) for the IKP was 22.0 ± 6.1 kg C m^− 2^ (0–100 cm) and 28.3 ± 3.2 kg C m^− 2^ (0–100 cm) for the FCR delta. Soil core N stocks varied from 0.5 to 1.78 kg N m^− 2^ for the first meter of soil and mean soil core SN stocks were 1.0 ± 0.4 and 1.5 ± 0.2 kg N m^− 2^ for the IKP and FCR delta, respectively.

**Table 1:** Soil organic carbon (SOC) and soil nitrogen (SN) stocks for the collected soil cores of the IKP and FCR delta for different reference depths. SOC stocks are given in kg C m^− 2^ and SN stocks are given in kg N m^− 2^. In addition, SOC and SN densities in kg C m^− 3^ and kg N m^− 3^ are provided in the supplementary material Table S2 and Table S3

Sample site	SOC 0–30 cm (kg C m^− 2^)	SOC 0–100 cm (kg C m^− 2^)	SOC 0–150 cm (kg C m^− 2^)	SOC 0–200 cm (kg C m^− 2^)	SN 0–30 cm (kg N m^− 2^)	SN 0–100 cm (kg N m^− 2^)	SN 0–150 cm (kg N m^− 2^)	SN 0–200 cm (kg N m^− 2^)	Core depth (cm)
IKP15-T1-0	7.70	16.54	27.16	49.82	0.28	0.46	1.02	2.37	195
IKP15-T1-1	4.92	16.61	31.30	51.59	0.27	0.98	1.62	2.61	200
IKP15-T1-2	8.48	18.84	26.49	45.78	0.24	0.75	1.12	2.20	199
IKP15-T1-3	11.42	26.39	33.82	44.52	0.56	1.43	1.71	2.47	209
IKP-DELT-1	7.70	31.77	39.82	42.47	0.44	1.46	1.98	2.09	201
**Mean**	8.05	22.03	31.72	46.84	0.36	1.02	1.49	2.35	
FCR-DELT-2	10.87	27.95	33.55	> 33.55^a^	0.49	1.25	1.49	> 1.49^a^	129
FCR-DELT-3	11.71	32.43	42.90	> 42.90^a^	0.69	1.78	2.31	> 2.31^a^	105
FCR-DELT-5a	10.25	24.59	27.26	29.67	0.61	1.57	1.70	1.87	157
FCR-DELT-5b	9.27				0.58				54
**Mean**	10.52	28.32	34.57	> 35.38^a^	0.59	1.53	1.83	> 1.89^a^	

^a^Minimum SOC and SN stocks for 0–200. SOC and SN stocks were only extrapolated 50 cm below the lowermost sample

At sites with sparse or no vegetation (IKP15-T1-0, IKP15-T1-1) SOC and SN stocks were higher in the deeper soil core portions below 100 cm. While both soil cores store around 17 kg C m^− 2^ in the first meter of soil they contained 33.3 and 35.0 kg C m^− 2^, respectively, from 100 to 200 cm indicating that a significant amount of C is buried below barren (sandy) deposits in the IKP delta. These numbers are especially important when considering the active layer thickness of 82 and 95 cm for IKP15-T1-0 and IKP15-T1-1. A major portion of the SOC is thus stored just below the depth of the current active layer extent.

### Radiocarbon dates and accumulation rates

A total of 24 samples were submitted for AMS radiocarbon dating (Table 2). Results indicate that SOC in the FCR delta is predominantly younger than 5000 years BP (except one age with 5530 ± 67 cal yr BP). The deposits in the IKP delta are even younger when not considering an age–depth inversion (IKP15-T1-3-9a; 6023 ± 99 cal yr BP) and one outlier (IKP-DELT-1-6; 10,046 ± 163 cal yr BP) with an early Holocene age. Omitting the two age–depth inversions (which were both bulk organic samples), core ages increase almost linearly with depth, pointing at a rather stable deposition environment.

**Table 2:** Dated samples and radiocarbon ages for the IKP and FCR deltas. Radiocarbon dates were calibrated with the Calib7.1 software [51, 52]. Post-bomb (modern) radiocarbon dates were calibrated with ^14^Chrono intcal13 [53, 54]. Modern radiocarbon dates indicate a negative calibrated age

Sample ID	Depth (cm)	Lab. no.	AMS ^14^C age (years BP)	Calib age (years BP)	Dated material	Coordinates	
						Latitude (°)	Longitude (°)
FCR-DELT-2-1	11–12	Poz-74742	235 ± 30 BP	292 ± 24	Moss leaves/stems	70.34799	− 151.38319
FCR-DELT-2-2	30–31	Poz-74743	925 ± 30 BP	852.5 ± 72	Moss leaves/stems	70.34799	− 151.38319
FCR-DELT-2-3	52–53	Poz-74744	1120 ± 30 BP	1022 ± 66	Sedge stems	70.34799	− 151.38319
FCR-DELT-2-4	99–100	Poz-74818	4780 ± 40 BP	5530 ± 67	Bulk organic (peat)	70.34799	− 151.38319
FCR-DELT-2-5	113–114	Poz-74819	2965 ± 35 BP	3116 ± 112	Bulk organic	70.34799	− 151.38319
FCR-DELT-3-1	20–21	Poz-74820	715 ± 30 BP	671.5 ± 26	Bulk organic	70.37390	− 151.34367
FCR-DELT-3-2	43–44	Poz-74821	2085 ± 30 BP	2067 ± 77	Moss leaves/stems	70.37390	− 151.34367
FCR-DELT-3-3	70–71	Poz-74822	2495 ± 30 BP	2608 ± 125	Moss leaves/stems	70.37390	− 151.34367
FCR-DELT-3-4	103–104	Poz-74823	2985 ± 30 BP	3156 ± 91	Moss leaves/stems	70.37390	− 151.34367
FCR-DELT-5a-1	17–18	Poz-74825	102.56 ± 0.31 pMC	− 6 ± 1	Sedge stems	70.38579	− 151.33944
FCR-DELT-5a-2	29–30	Poz-74826	450 ± 30 BP	503.5 ± 32	Sedge stems	70.38579	− 151.33944
FCR-DELT-5a-3	43–44	Poz-74827	1095 ± 30 BP	999 ± 61	Wood with bark	70.38579	− 151.33944
FCR-DELT-5a-4	98–99	Poz-74828	2065 ± 30 BP	2035 ± 85	Sedge stems	70.38579	− 151.33944
FCR-DELT-5a-5	131–133	Poz-74829	4425 ± 35 BP	4971 ± 97	Bulk organic	70.38579	− 151.33944
IKP-DELT-1-1	4–5	Poz-74830	112.76 ± 0.29 pMC	− 44 ± 1	Bulk organic	70.79137	− 154.43627
IKP-DELT-1-2	34–35	Poz-74831	345 ± 30 BP	399 ± 86	Moss leaves/stems	70.79137	− 154.43627
IKP-DELT-1-3	53–54	Poz-74968	735 ± 30 BP	683 ± 27	Moss leaves/stems	70.79137	− 154.43627
IKP-DELT-1-4	85.5–86.5	Poz-74756	1180 ± 30 BP	1115 ± 66	Moss leaves/stems	70.79137	− 154.43627
IKP-DELT-1-5	133.5–134.5	Poz-74757	1395 ± 30 BP	1315 ± 35	Sedge stems	70.79137	− 154.43627
IKP-DELT-1-6	199.5–200.5	Poz-74758	8910 ± 60 BP	10,046 ± 163	Bulk organic	70.79137	− 154.43627
IKP15-T1-3-2a	16–17	Poz-89355	103.41 ± 0.32 pMC	− 6 ± 1	Sedge stems	70.75350	− 154.46600
IKP15-T1-3-4a	45–47	Poz-89356	915 ± 30 BP	841 ± 79	Sedge stems	70.75350	− 154.46600
IKP15-T1-3-9a	149–150	Poz-89322	5250 ± 40 BP	6023 ± 99	Bulk organic	70.75350	− 154.46600
IKP15-T1-3-13a	215–216	Poz-89323	2185 ± 30 BP	2216.5 ± 94	Sedge stems	70.75350	− 154.46600

**Fig. 2:**
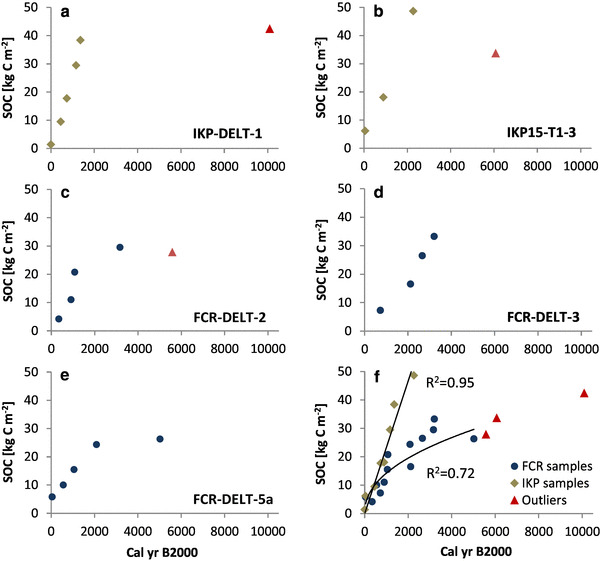
Calibrated radiocarbon dates in relation to cumulative SOC storage for five investigated soil cores in the IKP (**a, b**) and FCR deltas (**c–e**). Lower right image (**f**) is a compilation of all radiocarbon dates with best-fit curves for the IKP and the FCR data. Brown diamonds are samples from the IKP delta and blue dots are from the FCR delta. Red triangles indicate age–depth inversions (IKP15-T1-3 and FCR-DELT-2) or outliers (IKP-DELT-1). The SOC (kg C m^− 2^) is always the cumulative SOC to a particular depth calculated for 1 m^2^

Accordingly, there is a near-linear increase of organic C accumulation with age (Fig. 2; Table 3) for the IKP delta. Following this linear trend in Fig. 2f, the mean linear C accumulation rate for the IKP delta is 23.3 g C m^− 2^ year^− 1^. For the FCR delta samples, this near-linear trend only holds for samples younger than 1000 cal yr BP and the best-fit curve in Fig. 2f shows a flattening trend towards older samples indicating lower accumulation rates. Average C accumulation rates according to Fig. 2f for the FCR delta are 14.3 g C m^− 2^ year^− 1^ for the last 1000 cal yr BP, 9.8 g C m^− 2^ year^− 1^ for the last 2000 cal yr BP and 5.9 g C m^− 2^ year^− 1^ for the last 5000 cal yr BP.

**Table 3:** Sediment (sediment acc rate) and organic carbon accumulation rates (OC acc rate). Sediment accumulation rate is based on the depth of the sample (subtracting intra-sedimentary ice) and the calibrated radiocarbon date. Organic carbon accumulation rate is based on cumulative soil organic carbon (Cum SOC) storage at a specific depth and the calibrated radiocarbon date at the corresponding depth. Mean sediment and organic carbon accumulation rates are calculated in relation to the soil surface (depth = 0 cm and Cum SOC = 0 kg C m^− 2^). Episodic sediment and organic carbon accumulation rates are calculated in relation to the sample above a particular sample and reflect the rates in a particular episode of time

Sample ID	Age (cal year B2000)	Cum SOC (kg C m^− 2^)	Depth (cm)	Mean sediment acc rate (mm year^− 1^)	Episodic sediment acc rate (mm year^− 1^)	Mean OC acc rate (g C m^− 2^ year^− 1^)	Episodic OC acc rate (g C m^− 2^ year^− 1^)
FCR-DELT-2-1	342	4.21	11–12	0.12	0.12	12.32	12.32
FCR-DELT-2-2	902.5	11.06	30–31	0.12	0.12	12.25	12.21
FCR-DELT-2-3	1071.5	20.76	52–53	0.18	0.48	19.38	57.42
FCR-DELT-2-4a^a^	5580	27.90	99–100	na	na	na	na
FCR-DELT-2-5	3166	29.55	113–114	0.18	0.17	9.33	4.20
FCR-DELT-3-1	721.5	7.30	20–21	0.14	0.14	10.11	10.11
FCR-DELT-3-2	2116.5	16.55	43–44	0.11	0.10	7.82	6.63
FCR-DELT-3-3	2657.5	26.51	70–71	0.14	0.26	9.97	18.40
FCR-DELT-3-4	3206	33.30	103–104	0.17	0.32	10.39	12.39
FCR-DELT-5a-1	43.7	5.83	17–18	1.97	1.97	133.28	133.28
FCR-DELT-5a-2	553.5	10.08	29–30	0.27	0.12	18.21	8.34
FCR-DELT-5a-3	1049	15.52	43–44	0.21	0.15	14.80	10.98
FCR-DELT-5a-4	2085	24.36	98–99	0.24	0.27	11.68	8.53
FCR-DELT-5a-5	5020.5	26.35	131–133	0.14	0.07	5.25	0.68
IKP-DELT-1-1	5.7	1.44	4–5	4.64	4.64	254.68	254.68
IKP-DELT-1-2	448.5	9.52	34–35	0.44	0.39	21.22	18.23
IKP-DELT-1-3	733	17.80	53–54	0.42	0.38	24.28	29.11
IKP-DELT-1-4	1165	29.50	85.5–86.5	0.39	0.32	25.32	27.09
IKP-DELT-1-5	1364.5	38.42	133.5–134.5	0.53	1.40	28.15	44.70
IKP-DELT-1-6a^a^	10,096	42.47	199.5–200.5	na	na	na	na
IKP15-T1-3-2a	43.7	6.18	16–17	3.77	3.77	141.44	141.44
IKP15-T1-3-4a	891	18.06	45–47	0.46	0.29	20.27	14.01
IKP15-T1-3-9a^a^	6073	33.71	149–150	na	na	na	na
IKP15-T1-3-13a	2266.5	48.67	215–216	0.54	0.55	21.48	22.26

^a^No sediment and organic carbon accumulation rates were calculated for age–depth inversions and outliers

For the sediment accumulation, rates in the IKP delta are also higher than in the FCR delta with a mean rate for the two dated soil cores of 0.53 and 0.54 mm year^− 1^ when excluding the outlier (IKP-DELT-1-6) and the age–depth inversion (IKP15-T1-3-9a) (Table 3) The mean sediment accumulation rate for the complete depth of the three soil cores in the FCR delta is between 0.14 and 0.18 mm year^− 1^, excluding the age–depth inversion (FCR-DELT-2-4).

### Grain-size distribution

**Fig. 3:**
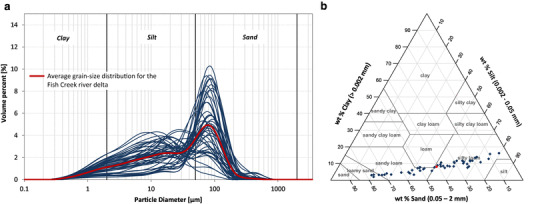
**a** Grain-size distribution for the FCR delta, red line indicates average grain-size distribution. **b** USDA soil texture triangle [58] with the analyzed samples (in wt%) from the FCR delta (blue diamonds). Red diamond shows the average soil texture for the FCR delta samples

**Fig. 4:**
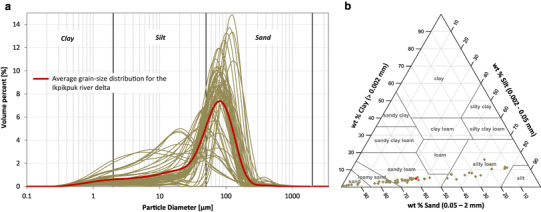
**a** Grain-size distribution for the IKP delta, red line indicates average grain-size distribution. **b** USDA soil texture triangle [58] with the analyzed samples (in wt%) from the IKP delta (brown diamonds). Red diamond shows the average soil texture for the IKP delta samples

Grain-size analysis shows small differences between the soil cores in the FCR and the IKP delta (Figs. 3a, 4a). Whereas the collected soil cores in the FCR delta consist more of medium to very coarse silt, the IKP delta soil cores contain more fine sand particles. In addition, grain-size distribution in the FCR delta is poorly to very poorly sorted (after Folk and Ward [63]) with a mix of unimodal and bimodal distribution curves. Bimodal distributed grain-size curves mostly have a peak in medium silt (8–16 µm) and a second, often major peak in very fine sand (63–125 µm). The grain-size distribution in the IKP delta is moderately to poorly sorted and shows a unimodal distribution in all except three analyzed samples (these three samples show a bimodal distribution) with the peak either in the very coarse silt or very fine sand fraction. An exception to this pattern is the core segment from 87 to 156 cm depth in IKP-DELT-1 which shows a distinct medium to coarse silt distribution with only a minor amount of very fine sand (< 17%). This core segment also includes the three bimodal distributed samples from the IKP delta. Common for all samples is the low amount of clay. Following the calculation in GRADSTATv8 [55], the main grain-size class (after Wentworth [64]) for the IKP delta is very coarse silt to fine sand and for the FCR delta is medium silt to very fine sand. Adapting to the U.S. Department of Agriculture (USDA) soil texture classification [58] the main soil texture type for the FCR delta is *‘*silty loam’ and for the IKP delta *‘*sandy loam’ (Figs. 3b, 4b).

### Arctic river delta C and N storage

Results for both upscaling approaches are in the same range for both SOC and SN estimations (Tables 4, 5). However, upscaling based on simple averaging had higher 95% confidence intervals and therefore a higher uncertainty. This is due to the fact of averaging C and N stocks of all soil permafrost cores across the different landscape units.

**Table 4:** Mean landscape SOC stocks for the two investigated Arctic river deltas. Results are presented for the different upscaling approaches and for all reference depths in kg C m^− 2^ ± 95% confidence intervals (CI) (calculated after Hugelius [65])

Soil organic carbon (kg C m^− 2^ ± 95% CI)	Ikpikpuk river delta			Fish Creek river delta		
	0–30 cm	0–100 cm	0–200 cm	0–30 cm	0–100 cm	0–200 cm
Average approach	7.6 ± 1.7	20.7 ± 5.0	44.0 ± 2.8	9.9 ± 0.8	26.6 ± 3.4	33.2 ± 7.2
Landsat 8 LCC	8.1 ± 0.5	20.1 ± 1.7	42.4 ± 1.6	9.1 ± 0.6	23.7 ± 2.4	37.9 ± 3.5

**Table 5:** Mean landscape SN stocks for the two investigated Arctic river deltas. Results are presented for the different upscaling approaches and for all reference depths in kg N m^− 2^ ± 95% confidence intervals (calculated after Hugelius [65])

Soil nitrogen (kg N m^− 2^ ± 95% CI)	Ikpikpuk river delta			Fish Creek river delta		
	0–30 cm	0–100 cm	0–200 cm	0–30 cm	0–100 cm	0–200 cm
Average approach	0.3 ± 0.1	1.0 ± 0.3	2.2 ± 0.1	0.6 ± 0.1	1.4 ± 0.2	1.8 ± 0.4
Landsat 8 LCC	0.4 ± 0.04	0.9 ± 0.1	2.1 ± 0.1	0.5 ± 0.1	1.2 ± 0.2	2.0 ± 0.2

**Fig. 5:**
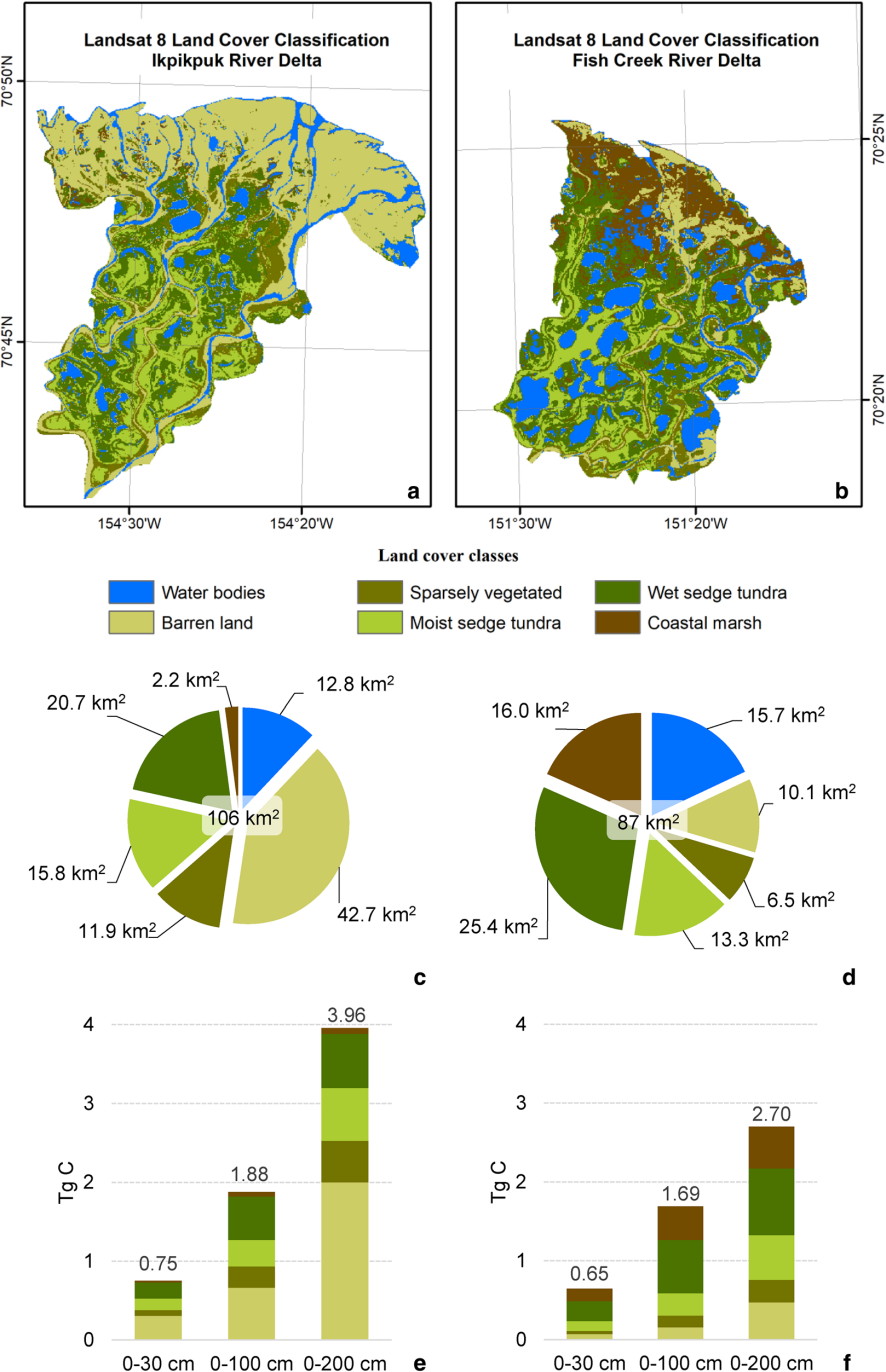
Top: LCC for the IKP (**a**) and FCR delta (**b**) based on Landsat 8 land cover classification. Middle: size of the land cover class areas for the IKP (**c**) and FCR (**d**) delta. Bottom: total river delta SOC (in Tg C) separated into the different land cover classes for the IKP (**e**) and FCR (**f**) delta

Our delta-wide mean SOC and SN stocks (± 95% confidence interval) based on the Landsat LCC (Fig. 5) for the first meter of soil are 20.1 ± 1.7 kg C m^− 2^ in the IKP delta and 23.7 ± 2.4 kg C m^− 2^ in the FCR delta (Table 4). This results in a total C storage for the deltas of 1.9 ± 0.2 Tg C for the IKP delta and 1.7 ± 0.2 Tg C for the FCR delta in the first meter of soil. However, there is a considerable amount of C stored in the second meter of soil. For 0–200 cm there is 4.0 ± 0.2 Tg C stored in the IKP delta and 2.7 ± 0.2 Tg C in the FCR delta. The difference between the two deltas when considering the entire upper 2 m of soil can be explained by the larger coverage of barren land in the IKP delta and the high C contents in the second meter of soil in barren lands.

Mean landscape SN storages based on the Landsat LCC for 0–100 cm are 0.9 ± 0.1 kg N m^− 2^ for the IKP and 1.2 ± 0.2 kg N m^− 2^ for the FCR delta (Table 5) leading to a total N stock of 0.08 ± 0.01 and 0.09 ± 0.01 Tg N for the IKP and FCR delta, respectively. For 0–200 cm, 0.34 ± 0.02 Tg N are stored in both deltas combined (0–200 cm). Similar to the organic C contents, there is a significant amount of N stored in the second meter of soil. The total C and N stocks for both deltas are presented in Table S4 in the supplementary material.

## Discussion

### Significance of carbon and nitrogen stocks in Arctic river deltas

Our results show the importance of Arctic river deltas as C and N rich permafrost deposits. Arctic river deltas, and thus their C stocks, are highly dynamic due to the abundant fluvial and thermokarst processes compared to other permafrost environments. The upscaled mean landscape stocks of 20.1 and 23.7 kg C m^− 2^ (0–100 cm) are in the same order compared to another study from deltaic deposits on the ACP by Ping et al. [19]. They sampled profiles on the Alaskan Beaufort Sea Coast including 13 exposures and soil pits in Arctic river deltas which resulted in a mean profile value of 29.7 ± 13.2 kg C m^− 2^ (0–100 cm). Similarly, C stocks from the Lena river delta studied by Zubrzycki et al. [22] were 29 ± 10 kg C m^− 2^ for the Holocene river terrace and 14 ± 7 kg C m^− 2^ for the active floodplain (0–100 cm). The latter, in particular, is similar to the sand-dominated profiles in our study, where IKP15-T1-0 and IKP15-T1-1 store ~ 17 kg C m^− 2^ in the first meter of soil. Also, Siewert et al. [17] found 9.6 ± 7 kg C m^− 2^ for alluvial sediment and 17.7 ± 5.8 kg C m^− 2^ for the floodplain on Kurungnakh Island in the Lena river delta.

However, the sometimes very high SOC stocks found on the ACP of Alaska are not present in the IKP and FCR delta. Average SOC stocks for the ACP are 62 kg C m^− 3^ (note that it is kg C m^− 3^) [9], 52 kg C m^− 3^ (0–100 cm) and 29 kg C m^− 3^ (> 100 cm) [66], and 50 kg C m^− 3^ in the Barrow area [67], 40 kg C m^− 3^ on the Barrow Peninsula [68], and 55.1 kg C m^− 2^ (0–100 cm) for Arctic lowlands of Alaska [11]. However, the above-mentioned studies focused more on peat-rich drained thaw lake basins instead of alluvial, silty sandy dominated deposits in Arctic river deltas. Nevertheless, peat-rich deposits also occur in Arctic river deltas. Ping et al. [19] sampled one exposure (40 cm) in the forefront of the IKP delta and found 32.8 kg C m^− 2^ in the upper peaty soil layer (0–40 cm). Based on five delta soil cores from the Mackenzie delta, Tarnocai et al. [6] calculated the SOC storage to be 65 kg C m^− 2^. Our results also indicate C rich deposits especially in the second meter of soil (see the following chapter), though no peat was cored in our sites.

There are not much data available on N storage in Arctic river deltas. Ping et al. [19] analyzed N in the delta cores of the Beaufort Sea Coast and found a mean profile N storage of 1.2 ± 0.8 kg N m^− 2^ (0–100 cm) and Zubrzycki et al. [22] analyzed soil cores in the Lena river delta with mean values of 1.2 kg N m^− 2^ for the Holocene river terrace and 0.9 kg N m^− 2^ for the active floodplain. These are very similar to our landscape mean values of 0.9 ± 0.1 kg N m^− 2^ (IKP) and 1.2 ± 0.2 kg N m^− 2^ (FCR). Overall, these river delta values are slightly lower than N stocks in other Arctic tundra environments (e.g., Obu et al. [23], Fuchs et al. [24]) which can be explained by the larger fraction of sandy deposits and sand-dominated texture in the cores in general. In many cases, the sand-dominated samples in our study had N contents below 0.1%, which was below the detection limit of the analyzing device (Vario EL III Elemental Analyzer). Nevertheless, the N stocks found in our study might become important when permafrost thaws and active layer deepens, leading to an increase of plant-available N. This could enhance plant growth, since nitrogen is one of the important limiting factors for vegetation growth in tundra environments [29–33].

### SOC and SN distribution with depth

The 200 cm soil cores allow a detailed analysis of C and N in the upper 2 m of the soil column. This turned out to be important, because especially in sandy deposits large amounts of C were stored in depths exceeding 1 m. This fits well with our understanding that Arctic deltas are, in addition to the yedoma domain [69] and deep peatlands [7], storing a substantial amount of frozen C and N below the active layer.

**Fig. 6:**
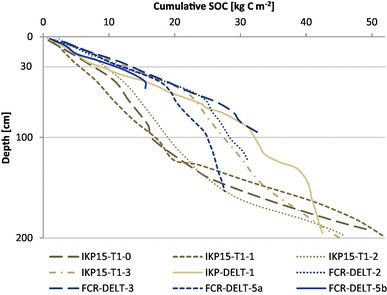
Cumulative SOC storage for the FCR and IKP river delta soil cores. Brown lines indicate the IKP cores, blue lines symbolize FCR cores

**Fig. 7:**
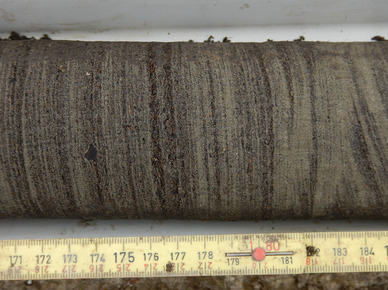
Peaty-silty to sandy permafrost core segment of IKP15-T1-1, at 171–184 cm depth, with densely laminated accumulation layers of alternating peat and silty sand layers in 1–5 mm intervals. Grain-size analysis (which excludes the peat/organic layers) indicated ‘sandy loam’ for this segment, according to the USDA soil texture classification [58], Photo: M. Fuchs, 15 July 2015

Cumulative SOC storage with depth for the collected soil cores (Fig. 6) indicates that some of the cores in the IKP delta contain a considerable amount of C below 100 cm. The reason for the significant increase in cumulative C in these cores is the presence of buried peaty layers in greater core depths. Both IKP15-T1-0 and IKP15-T1-1 are characterized by a thick sandy active layer. But below the first meter these sandy deposits include abundant peat inclusions and silt layers. Figure 7 shows a subsample of IKP15-T1-1 with dense laminations of peaty layers alternating with sandy silty layers in 1–5 mm intervals. This fine lamination appears also in other cores (e.g., IKP15-T1-2) in the IKP delta at different depths and can be interpreted as originating from periodic (possibly even annual) river flooding in a low-energy backwater setting. These soil cores (IKP15-T1-0, IKP15-T1-1, IKP15-T1-2) show increased C and N storage below 100 cm (Fig. 6). In the IKP15-T1-0 and IKP15-T1-1 cores, 67 and 68%, respectively of the SOC are stored below 100 cm depth, while the active layer thickness at these sites was 82 and 95 cm, respectively. Bockheim and Hinkel [66] indicated the importance of deeper soil layers for the permafrost C pool in drained lake basins. But in comparison to Bockheim and Hinkel [66], where the second meter of soil (typical frozen lake sediments) contributes 36% to the organic C in the first 2 m, cores in the IKP delta show that on average 52% of the organic C stored in the first 2 m of soil occur below 100 cm. The rather inhomogeneous or even increasing distribution of C with depth is indicative of the importance of the deltaic depositional environment rather than soil forming processes for SOC storage in the soils. The same is true for SN in the IKP delta where 60% of SN is stored in the second meter of soil. The FCR delta cores do not show increasing C or N contents with increasing depth; however, the analyzed soil cores were all shallower than 2 m. Nevertheless, in both study sites combined, 46% of organic C and 51% of N are stored in the 100–200 cm interval.

The high heterogeneity of C and N in deltaic soil cores and the potentially large thickness of Arctic river delta deposits highlight the significance of including deeper soil permafrost cores from Arctic river deltas in future C and N stock estimation studies. The knowledge of the depth of alluvium is in general very limited for Arctic river deltas [7]. More data would help to estimate the entire SOC and SN storage in Arctic river delta deposits. For example, in the Colville river delta, Jorgenson et al. [5] estimated that sandy deposits reach down to 5–10 m below sea level, followed by 6–12 m of gravelly material and 20 m or more of interbedded silts, clays and organics. This results in at least 31 m of deltaic deposits below modern sea level. Different studies from the Mackenzie Delta show depth of alluvium between 30 and 58 m (e.g., Johnston and Brown [70], Taylor et al. [71], Smith et al. [72]) and Schwamborn et al. [73, 74] estimated the depth of the fluvial sandy Arga Complex in the Lena Delta to a minimum of 60 m. Therefore, Arctic river delta deposits contain significant amounts of frozen C, considering the great depth of alluvium.

A simplified organic C quantification, may be the integration of an average depth of alluvium of 30 m with our mean SOC storage of the deltas [42.4 kg C m^− 2^ for the IKP and 37.9 kg C m^− 2^ for the FCR (0–200 cm)]. This would result in a C stock of 67.5 Tg C for the IKP and 49.4 Tg C for the FCR delta if the deposits are 30 m thick. This estimate leads to a total organic C stock of 117 Tg (~ 20 kg C m^− 3^) for both deltas combined. The two studied deltas cover less than 200 km^2^ and are not considered in the below 3 m SOC estimation for deltaic deposits in the Northern Circumpolar Soil Carbon Database (NCSCD) by Hugelius et al. [7]. In the NCSCD, the deltaic mean SOC content for the stocks below 3 m depth are estimated to include 26.0 kg C m^− 3^ [7], which is similar to the results in our study.

With including small Arctic river deltas in circum-polar C estimates, current 0–3 m estimates will probably become slightly lower compared to estimates by Tarnocai et al. [6] and Hugelius et al. [7], because more barren ground covers the surface in river deltas compared to vegetated tundra landscapes. This shows the comparison of the SOC values in our study with the NCSCD, where the IKP and FCR deltas are part of large regional polygons (14,233 and 7361 km^2^) covering vast areas of the Arctic Coastal Plain. The polygon including the IKP delta is estimated to store 27.6 kg C m^− 2^ and the polygon including the FCR delta stores 73.3 kg C m^− 2^ (0–100 cm) [7]. This shows that data from small permafrost environments can improve the accuracy of circum-Arctic C estimations. In addition, the inclusion of small Arctic river deltas will increase the below 3 m C estimations, because these areas have not been covered as deltaic deposits in previous studies where, e.g., Tarnocai et al. [6] included seven and Hugelius et al. [7] included 12 major Arctic river deltas. Therefore, not only additional soil data from river deltas but also a better understanding of the spatial extent and depth of sediment deposits of small Arctic river deltas is needed for a more accurate estimation on deltaic C stocks.

### Sedimentary characteristics

#### Accumulation rates

The sediment accumulation rates are two to three times higher for the IKP delta compared with the FCR delta. Whereas both rivers drain parts of the Ikpikpuk Sand Sea [44], which is a source for sandy material, a possible reason for this difference is the different sizes of the catchments. The IKP drains a catchment that is three times bigger and reaches a higher altitude compared to the FCR catchment. This enables a higher sediment supply. In combination with the mean river discharge which is more than four times higher in the IKP than in the FCR [46, 47], this certainly has an impact on transport of sediments and deposition dynamics in the deltas.

Sediment accumulation rates found in our study area compare well with rates reported by Jorgenson et al. [3] for the Colville Delta of 2.6 mm year^− 1^ for delta active-floodplain cover deposits, 0.4 mm year^− 1^ for delta abandoned-floodplain cover deposits, and 1.3 mm year^− 1^ for delta inactive floodplain cover deposits. However, these rates include not only the net sediment gain but include ice within the sediment as well and are not based on radiocarbon dates but on direct measurements.

Also, mean organic C accumulation rates are higher in the IKP delta and range from 21.5 to 28.2 g C m^− 2^ year^− 1^ compared to 5.3 to 10.4 g C m^− 2^ year^− 1^ for the FCR delta. These accumulation rates are in the same range as what has been reported by Fuchs et al. [24] (2.7–49.7 g C m^− 2^ year^− 1^ for the Holocene) for thermokarst-affected landscapes in Arctic Siberia. However, compared to these areas, Arctic river deltas are more dynamic, because they are not only influenced by accumulation but also by erosion from ice break-up and spring flood dynamics.

For the calculation of mean soil organic C and sediment accumulation rates, three samples were excluded which were either age–depth inversions (FCR-DELT-2-4; IKP15-T1-3-9a) or an outlier (IKP-DELT-1-6). All three samples were bulk organic samples and therefore may be an indicator of older material being eroded and re-transported from permafrost cutbanks in the catchment. This dispersed organic matter litter may have been transported over long distances and finally deposited in the river delta. Nelson et al. [75] described a similar case when dating bulk organic samples. Fine organic material that is reworked from Pleistocene-age sediments lead to ages too old in their study on samples from an exposure on the Ikpikpuk River. In another study Stanley [76] reported problems with radiocarbon dating in modern river deltas as sediment gets reworked, often leading to age inversions. Nevertheless, our results in general show a good age–depth relationship, not only in single cores but also when comparing different cores from the same river delta (see Fig. 2f), suggesting that the derived rates are indeed realistic.

#### Sediment distribution

The grain-size distribution for the IKP and FCR samples indicates slight differences in sediment source, transport, and depositional environments between the two deltas. All samples (except three) from the IKP Delta are unimodal and poorly to moderately sorted with a peak mostly in the sand fraction, indicating that the dominating transport mechanism is flowing water. Most of the material has been deposited by the river, even though some samples have a peak in coarse silt and are classified as silty loam. But in a braided river system like the IKP River, the stream flow might be low enough to deposit coarser silt. Most of the soil cores in the IKP delta are close to a side arm of the IKP River and far from the main channel. The more sand-dominated environment of the IKP delta is also visible in the LCC (see Fig. 5). The Landsat 8 LCC for the IKP delta reveals that 52% of the delta area is covered by sand (barren land) or only sparsely vegetated ground in contrast to the FCR delta classification, where only 19% is covered by barren or sparsely vegetated ground.

FCR delta soil texture shows more samples with bimodal distribution with a peak in the silt and a peak in the sandy fraction but mostly very poorly sorted. This mix of unimodal and bimodal distribution is an indication that sediments may have not only been deposited by the river but also through alluvial and lacustrine processes. For example, ponding water can act as traps for aeolian sediment. Migrating river channels can lead to abandoned river channels or periodically flooded lakes and ponds in the backwater which then act as sediment traps where fine-grained material can settle. A mixed grain-size signal in the cores indicates thus the variability and changing nature of migrating river channels.

Both river deltas are similar in size, but they differ in grain-size composition and accumulation rates which both might be an effect of the different peak discharges and the different characteristics of the two watersheds which the Ikpikpuk and Fish Creek river drain. Moreover, even though the SOC and SN socks are in the same range, there is not a uniform distribution of C and N with depth. This shows the need for more soil C data in Arctic river deltas, since these areas are highly heterogeneous.

The migration of delta channels, spring floods, tidal regimes, and thermokarst processes lead to a complex mixture of different depositional environments, which to some extent is reflected in the heterogeneous soil cores. Also, these deltas are part of a large tundra system which is shaped by these rivers during the Holocene and knowledge of these meanders and braiding patters would increase our understanding of present day processes.

### Impacts of future changes

Our study indicates the size and importance of C and N pools in Arctic river deltas and demonstrates the heterogeneity of these environments. With continued climate warming in the Arctic, these environments at the land–ocean interface will increasingly be affected by permafrost thaw, increased coastal erosion, and sea level rise, which in turn will also affect the deep deltaic C rich deposits of small Arctic river deltas such as the IKP and FCR delta. Currently, a study from 2005 by Jorgenson and Brown [77] found that the IKP delta had a mean negative erosion rate of − 0.4 m year^− 1^, so it expands, whereas the FCR delta had a mean erosion rate of 2.5 m year^− 1^. The same study [77] also states that assessing erosion rates and C inputs for Arctic river deltas are difficult due to their heterogeneous and complex characteristic. Ping et al. [19] found that erosion rates in studied Alaskan Arctic river deltas generally are small and that the deltas often have accreting shorelines. Increased permafrost thaw and erosion may even result in increased sediment loading in these rivers over the short term [78–80]. However, as sea level is projected to rise substantially [81], it is highly questionable whether small Arctic delta accretion, based on our estimates of slow sediment accumulation rates, will keep up with the pace of sea level rise. It can be expected that the shallowest parts of the coast will be affected by inundation with ongoing sea level rise, affecting SOC and SN stocks in current permafrost deposits in the IKP and FCR delta.

### Significance of remotely sensed upscaling results

Our approach is a straight-forward, first-order attempt to classify the landscape into the most dominant land cover classes based on spatially weighted upscaling. We think that such an approach is a reasonable and logical attempt to go beyond averaging the total C stocks only based on mean soil core SOC contents and no landscape information. The 95% confidence intervals are smaller with the Landsat 8 based upscaling, in comparison with a simple average approach, showing that a land cover-based upscaling improves the stock estimation result.

The accuracy assessment of the Landsat 8 based LCC showed an overall agreement for the classification of 78% and a kappa index of agreement of 0.73. Especially for the more diverse FCR delta, a higher resolution land cover map in combination with more soil data would be beneficial to capture its heterogeneity. In particular, soil permafrost cores from the land cover class ‘Coastal marsh’ which was not sampled, would help to improve the upscaling. Our LCC solely focuses on the two river deltas and due to its focus on only six classes it is more suitable for an upscaling based on the amount of collected soil cores in comparison with the more diverse map of the NSSI [43] and has a higher spatial resolution than a previous mapping approach of the Alaska North Slope, e.g. [38]. Deeper cores exceeding 100 or even 300 cm and information about thickness of deltaic alluvium deposits would further increase the accuracy of SOC and SN stock estimation and lead to a better understanding of C pools in Arctic river deltas.

## Conclusion

River deltas are at the interface of the terrestrial and marine ecosystems. Thus, the complexity of Arctic river deltas is caused by cold-climate fluvial, coastal, marine, and permafrost-related ground ice aggradation and degradation processes, all of which affect soil C and N stocks of deltaic deposits. Our study presents SOC and SN stock estimations as well as organic C and sediment accumulation rates for two small Arctic river deltas on the ACP of northern Alaska. Based on scaling the permafrost core results with remotely sensed images, the mean landscape-scale SOC storage is 20.1 ± 1.7 and 23.7 ± 2.4 kg C m^− 2^ for the first meter of soil and SN storage is 0.9 ± 0.1 and 1.2 ± 0.2 kg N m^− 2^ (0–100 cm) for the IKP and FCR deltas, respectively. In addition, there is more SOC (2.1 Tg C) and SN (0.12 Tg N) stored in the second meter of soil in the IKP delta revealing the importance to include deeper cores in future C stock estimations. In total, 6.7 Tg C and 0.34 Tg N are stored in the first 2 m of the IKP and FCR delta combined.

The organic C accumulation rates indicate differences in the two deltas. Our analysis shows that the IKP delta has in average a C accumulation rate of 23.3 g C m^− 2^ year^− 1^, while the FCR delta cores indicate lower rates with an average C accumulation rate of 9.8 g C m^− 2^ year^− 1^ for the past 2000 cal yr BP, likely reflecting slightly different watersheds and depositional characteristics of the two deltas. Whereas the IKP delta is dominated by a high amount of barren ground and sandy deposits, the FCR delta consists of more silt-dominated tundra landscapes. Our study demonstrates that small Arctic river deltas have to be considered in future permafrost C sock estimations, as already the two investigated deltas with a combined size of just < 200 km^2^ may store more than 100 Tg C. This indicates that even small river deltas contribute to the total C and therefore more data from these environments will improve future circum-Arctic C estimations. Thus, small Arctic river deltas have to be considered as C and N rich permafrost environments that are highly dynamic and vulnerable to future changes driven by a rapidly warming Arctic.

### Data availability

The data presented in this article is available online on the PANGAEA data repository (10.1594/PANGAEA.890883).

## Electronic supplementary material

Below is the link to the electronic supplementary material.

Supplementary material 1 (PDF 1827 KB)

## Footnotes

1 These twelve deltas include the Lena, Mackenzie, Yana, Indigirka, Yenisey, Kolyma, Ob, Pechora, Yukon, Pyasina, Colville and Olenek river deltas which cover an area of 75,800 km^2^ (Hugelius et al. [7]).
