# Supplementary material for: Sedimentary and geochemical characteristics of two small permafrost-dominated Arctic river deltas in northern Alaska
Source: Arktos. 2018 Jun 22;4(1):1–18. doi: 10.1007/s41063-018-0056-9 (PMC7659425; doi:10.1007/s41063-018-0056-9)
Supplement: Supplementary file 1 — Supplementary material 1 (PDF 1827 KB) [file 41063_2018_56_MOESM1_ESM.pdf]

Supplementary Material to:

Sedimentary and geochemical characteristics of two small permafrost-dominated Arctic river deltas in northern Alaska

Matthias Fuchs[1,2], Guido Grosse[1,2], Benjamin M. Jones[3], Jens Strauss[1], Carson A. Baughman[4], and Donald A. Walker[5]

[1]Alfred Wegener Institute Helmholtz Centre for Polar and Marine Research, Telegrafenberg A45, 14473 Potsdam, Germany
[2]Institute of Earth and Environmental Sciences, University of Potsdam, Karl-Liebknecht-Str. 24-25, 14467 Potsdam, Germany
[3]Water and Environmental Research Center, University of Alaska Fairbanks, 437 Duckering, PO Box 755860, Fairbanks, AK 99775, USA
[4]U.S. Geological Survey, Alaska Science Center, 4210 University Drive, Anchorage, AK 99508, USA.
[5]Alaska Geobotany Center, Institute of Arctic Biology, University of Alaska Fairbanks, 311 Irving, PO Box 757000, Fairbanks, AK 99775, USA.

Correspondence to: Matthias Fuchs (matthias.fuchs@awi.de)

Description of contents of the supplementary material

This supplementary material for the article *"Sedimentary and geochemical characteristics of two small permafrost dominated Arctic river deltas in northern Alaska"* presents additional soil core information, complementary maps, and consists of four tables and six figures.

Core characteristics

The sample locations including the basic permafrost core properties, the sample date, the core depth as well as organic layer and active layer thickness are presented in Table S1. In addition, Figure S1 shows the coring locations in both deltas with two close-up images from the Ikpikpuk sample sites (TES15-T1) in July 2015. In addition, soil organic carbon (SOC) and soil nitrogen (SN) densities in kg C m^{-3} and kg N m^{-3} are presented in Table S2 and S3 as supplement to Table 1 of the main article.

Total carbon and nitrogen stocks

In addition to the mean landscape (SOC) and (SN) contents presented in Table 4 and 5 in the main article, Table S4 shows the total SOC and SN stocks (in Tg) for the Ikpikpuk and Fish Creek river delta for the reference depths 0-30 cm, 0-100 cm, and 0-200 cm. These SOC and SN stocks are calculated

based on the methods described in chapter 3.4 *Scaling soil core carbon and nitrogen stocks to landscape level*.

Geochemical results

With Figure S2, we present the results of the geochemical analysis of the nine permafrost cores (in total 129 samples) in more detail. This includes the soil organic carbon content (SOCC) and the soil nitrogen content (SNC) which were calculated by multiplying the total organic carbon (TOC) with the dry bulk density, or in case of the SNC, the total nitrogen (TN) with the dry bulk density. In addition, Figure S2 shows the carbon to nitrogen (C/N) ratio (quotient of TOC and TN), the volumetric ice content (quotient of volume of ice in a sample and total volume of a sample) and the grain-size analysis results. The grain size results are presented in a line diagram, where the clay fraction is illustrated in a black line, the silt fraction in a brown line, and the sand fraction in a grey line. Grain size is displayed in volume percentage where the sum of clay, silt and sand adds up to 100%.

Supplementary grain-size data interpretation

Figure S3 presents a comparison of Figure 3 and 4 from the main article. In Figure S3, the average grain-size distributions for both river deltas is presented. The blue line shows the average grain-size distribution for the Fish Creek river delta and the brown line shows the average grain-size distribution for the Ikpikpuk river delta.

An additional analysis based on the relationship of grain-size distribution and the TOC respectively TN results is presented in Figure S4. The grain-size fraction of each analyzed sample was put in relation to the TOC and TN to check if there is a correlation between the grain-size and the organic carbon and nitrogen content within a sample. In general, the higher the sandy fraction in a sample, the less organic carbon and nitrogen is in a particular sample. The relationship between grain-size and total nitrogen is stronger than between grain-size and TOC in the analyzed samples. The best fit curves (red lines in Fig. S4) show the strongest relationship between the clay content and the carbon and nitrogen content in the samples. However, we only analyzed samples with a clay content up to 15%; therefore, this relationship is only valid for samples with a clay content up to 15%.

Watershed analysis

The watershed of the Ikpikpuk and Fish Creek river were calculated based on a pan-arctic digital elevation model with a ~90 m spatial resolution [1]. This analysis was carried out in ArcGIS 10.4 with the Hydrology toolbox according to Jones et al. [2]. In an additional step, the watershed was characterized in more detail by intersecting it with the surficial deposit classes by Jorgenson et al. [3].

The watershed of the Ikpikpuk River (Figure S5) has a spatial extent of 15,330 km^2 with the highest point at 468 m. a.s.l. The Fish Creek River watershed is significantly smaller covering an area

of 4,815 km^2 with the highest point at an altitude of 336 m. a.s.l. Both rivers drain a significant part of the Ikpikpuk sand sea, which consists of stabilized Pleistocene dune fields [4,5]. The partitioning of the watershed into the different surficial deposit classes is presented in Figure S6.

Table S1 Locations and general soil core characteristics for the Ikpikpuk river (IKP) delta and the Fish Creek river (FCR) delta sample site. Mean organic layer (OL) and mean active layer (AL) depth are in cm. Latitude and Longitude are in WGS84 in decimal degrees (°). Mean active layer depths are not available (na) for the cores collected in April 2014.

**Sample site**	**Latitude [°]**	**Longitude [°]**	**Sampling date**	**Mean OL depth [cm]**	**Mean AL depth [cm]**	**Core depth [cm]**	**Land cover class**
IKP15-T1-0	70.7549	-154.4843	15.07.2015	0	79	195	Barren land
IKP15-T1-1	70.7545	-154.4840	15.07.2015	0	96	200	Sparsely vegetated
IKP15-T1-2	70.7535	-154.4820	15.07.2015	3	49	199	Moist sedge tundra
IKP15-T1-3	70.7523	-154.4789	15.07.2015	17	42	209	Moist sedge tundra
IKP-DELT-1	70.7914	-154.4363	19.04.2014	1	na	201	Sparsely vegetated
FCR-DELT-2	70.3480	-151.3832	14.04.2014	53	na	129	Wet sedge tundra
FCR-DELT-3	70.3739	-151.3437	14.04.2014	3	na	105	Wet sedge tundra
FCR-DELT-5a	70.3858	-151.3394	15.04.2014	18	na	157	Wet sedge tundra
FCR-DELT-5b	70.3858	-151.3394	15.04.2014	4	na	54	Wet sedge tundra

Table S2 Soil organic carbon (SOC) densities for the collected soil cores of the IKP and FCR delta for different reference depths. This is in supplement to Table 1 of the main article.

**Sample site**	**SOC 0-30 cm**	**SOC 0-100 cm**	**SOC 0-150 cm**	**SOC 0-200 cm**	**Core depth**
	kg C m^{-3}	kg C m^{-3}	kg C m^{-3}	kg C m^{-3}	cm
**IKP15-T1-0**	25.67	16.54	18.11	24.91	195
**IKP15-T1-1**	16.40	16.61	20.87	25.80	200
**IKP15-T1-2**	28.27	18.84	17.66	22.89	199
**IKP15-T1-3**	38.07	26.39	22.55	22.26	209
**IKP-DELT-1**	25.67	31.77	26.55	21.24	201
**Mean**	26.81	22.03	21.15	23.42	
**FCR-DELT-2**	36.23	27.95	22.37		129
**FCR-DELT-3**	39.03	32.43	28.60		105
**FCR-DELT-5a**	34.17	24.59	18.17	14.84	157
**FCR-DELT-5b**	30.90	Ice wedge below 54 cm			54
**Mean**	35.08	28.32	23.05	14.84	

Table S3 Soil nitrogen (SN) densities for the collected soil cores of the IKP and FCR delta for different reference depths. This is in supplement to Table 1 of the main article.

**Sample site**	**SN 0-30 cm**	**SN 0-100 cm**	**SN 0-150 cm**	**SN 0-200 cm**	**Core depth**
	kg N m^{-3}	kg N m^{-3}	kg N m^{-3}	kg N m^{-3}	cm
**IKP15-T1-0**	0.93	0.46	0.68	1.19	195
**IKP15-T1-1**	0.90	0.98	1.08	1.31	200
**IKP15-T1-2**	0.80	0.75	0.75	1.10	199
**IKP15-T1-3**	1.87	1.43	1.14	1.24	209
**IKP-DELT-1**	1.47	1.46	1.32	1.05	201
**Mean**	1.19	1.02	0.99	1.17	
**FCR-DELT-2**	1.63	1.25	0.99		129
**FCR-DELT-3**	2.30	1.78	1.54		105
**FCR-DELT-5a**	2.03	1.57	1.13	0.94	157
**FCR-DELT-5b**	1.93	Ice wedge below 54 cm			54
**Mean**	1.98	1.53	1.22	0.94	

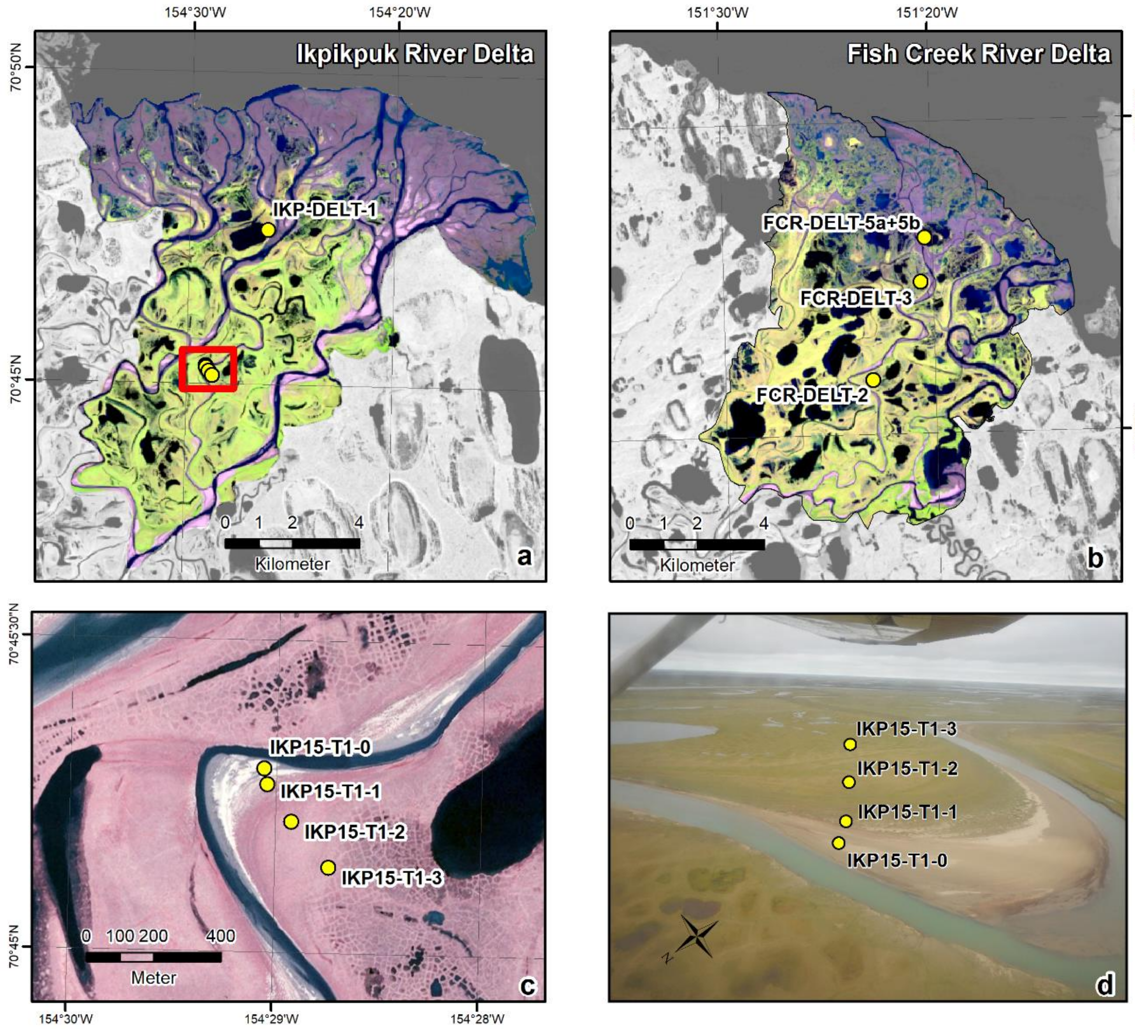

Figure S1 Soil core locations in (**a**) Ikpikpuk river delta (Landsat 8, acquisition date: 5 August 2016), and (**b**) Fish Creek river delta (Landsat 8, acquisition date: 5 August 2016). (**c**) Close-up of the Ikpikpuk river delta transect on a false-color ortho-photo (ID: DI00000100016777, U.S. Geological Survey, DOQ, Earth Explorer) and (**d**) photography of the close-up in (**c**) (Photo: M. Fuchs, 15.7.2015).

Table S4 Total soil organic carbon (SOC) and soil nitrogen (SN) stocks in teragram (Tg) for the Ikpikpuk and Fish Creek River deltas. Results are presented for two upscaling approaches (Landsat 8 and Average Approach) as well as for the different reference depths.

**Total SOC**	**Ikpikpuk river delta**			**Fish Creek river delta**		
	0-30 cm	0-100 cm	0-200 cm	0-30 cm	0-100 cm	0-200 cm
Average Approach	0.71 Tg C	1.93 Tg C	4.11 Tg C	0.71 Tg C	1.90 Tg C	2.37 Tg C
Landsat 8 LCC	0.75 Tg C	1.88 Tg C	3.96 Tg C	0.65 Tg C	1.69 Tg C	2.70 Tg C
**Total SN**	**Ikpikpuk river delta**			**Fish Creek river delta**		
	0-30 cm	0-100 cm	0-200 cm	0-30 cm	0-100 cm	0-200 cm
Average Approach	0.03 Tg N	0.09 Tg N	0.21 Tg N	0.04 Tg N	0.10 Tg N	0.13 Tg N
Landsat 8 LCC	0.03 Tg N	0.08 Tg N	0.20 Tg N	0.03 Tg N	0.09 Tg N	0.14 Tg N

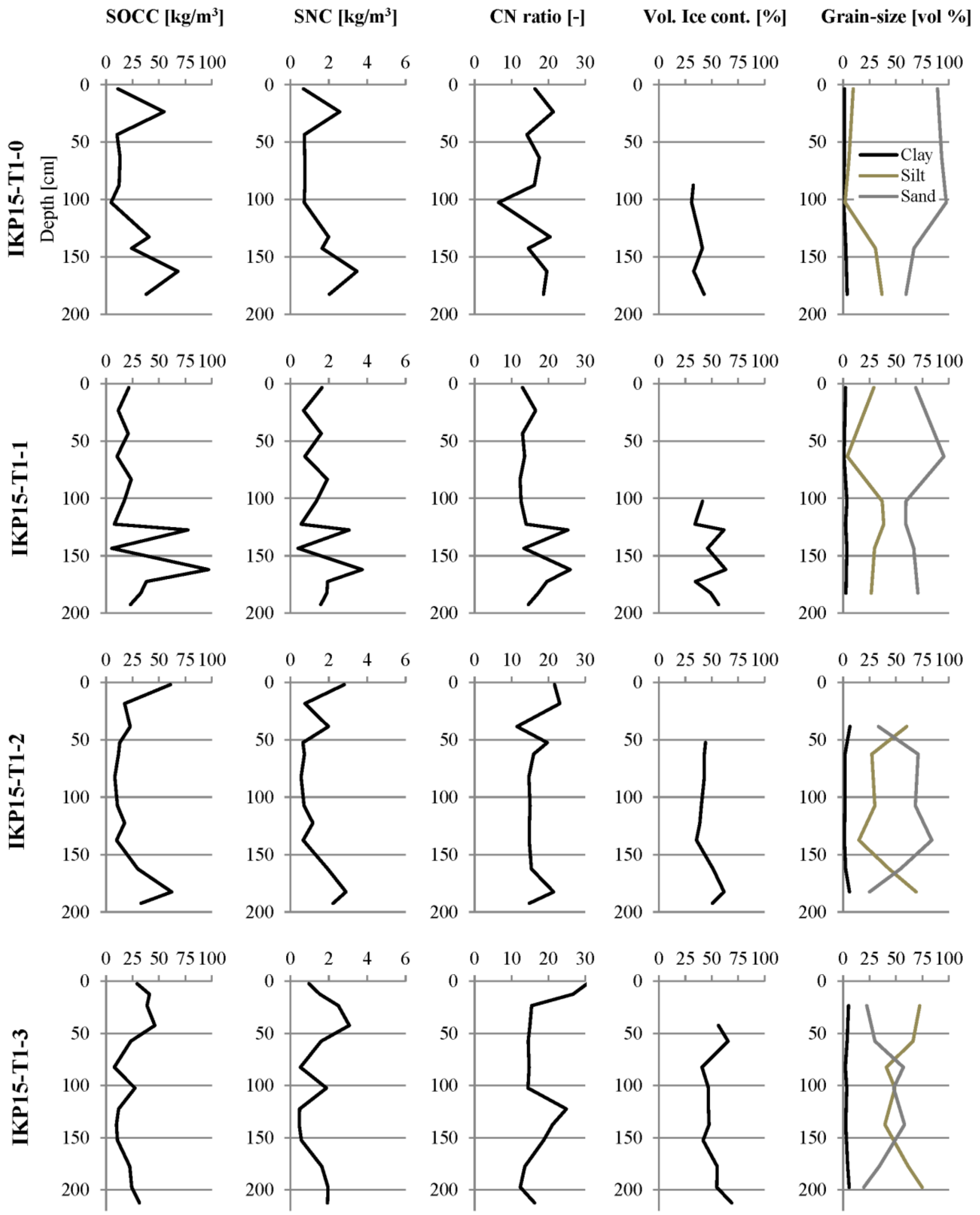

Figure S2 Soil sample properties for the nine studied core profiles. SOCC: soil organic carbon content in kg/m^3; SNC: soil nitrogen content in kg/m^3; CN ratio: carbon-nitrogen ratio; Vol. ice content: volumetric ice content in %; Grain-size: grain-size distribution in volume %, black line shows the % clay, brown line shows the % silt, and gray line shows % sand within an analyzed sample.

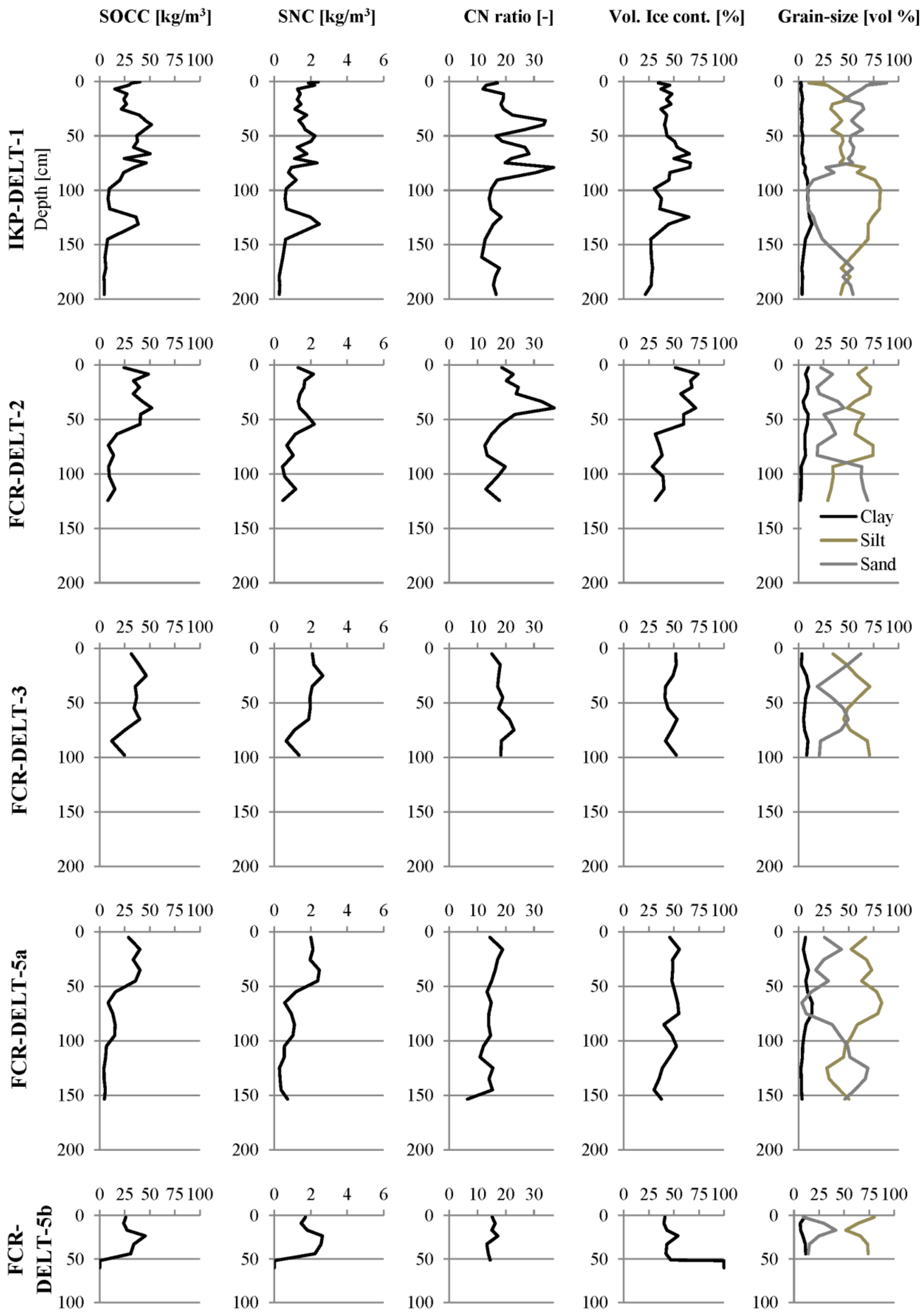

Figure S2 continued

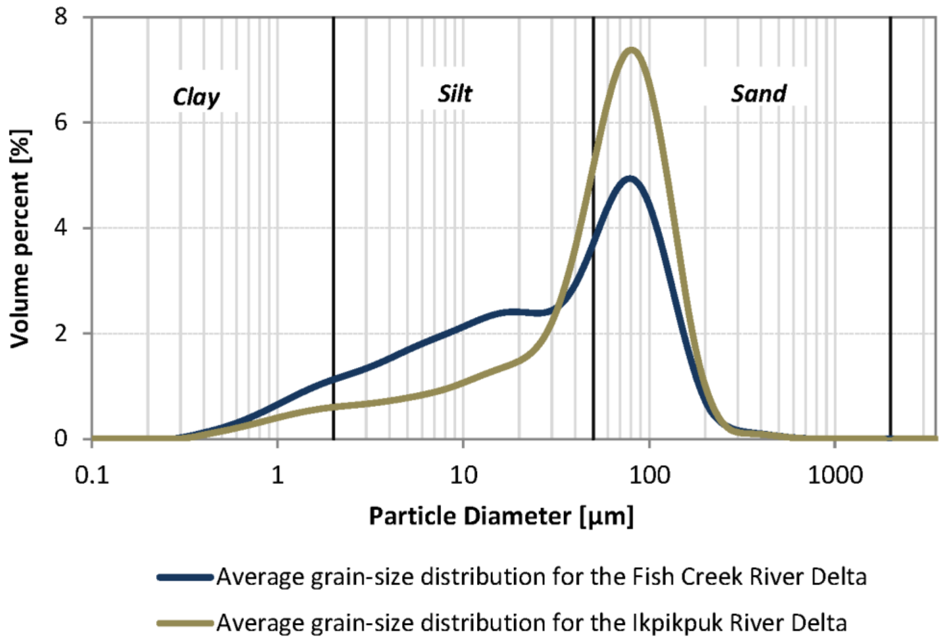

Figure S3 Average grain-size distribution for the FCR (n=48 samples) and IKP (n=56 samples) delta.

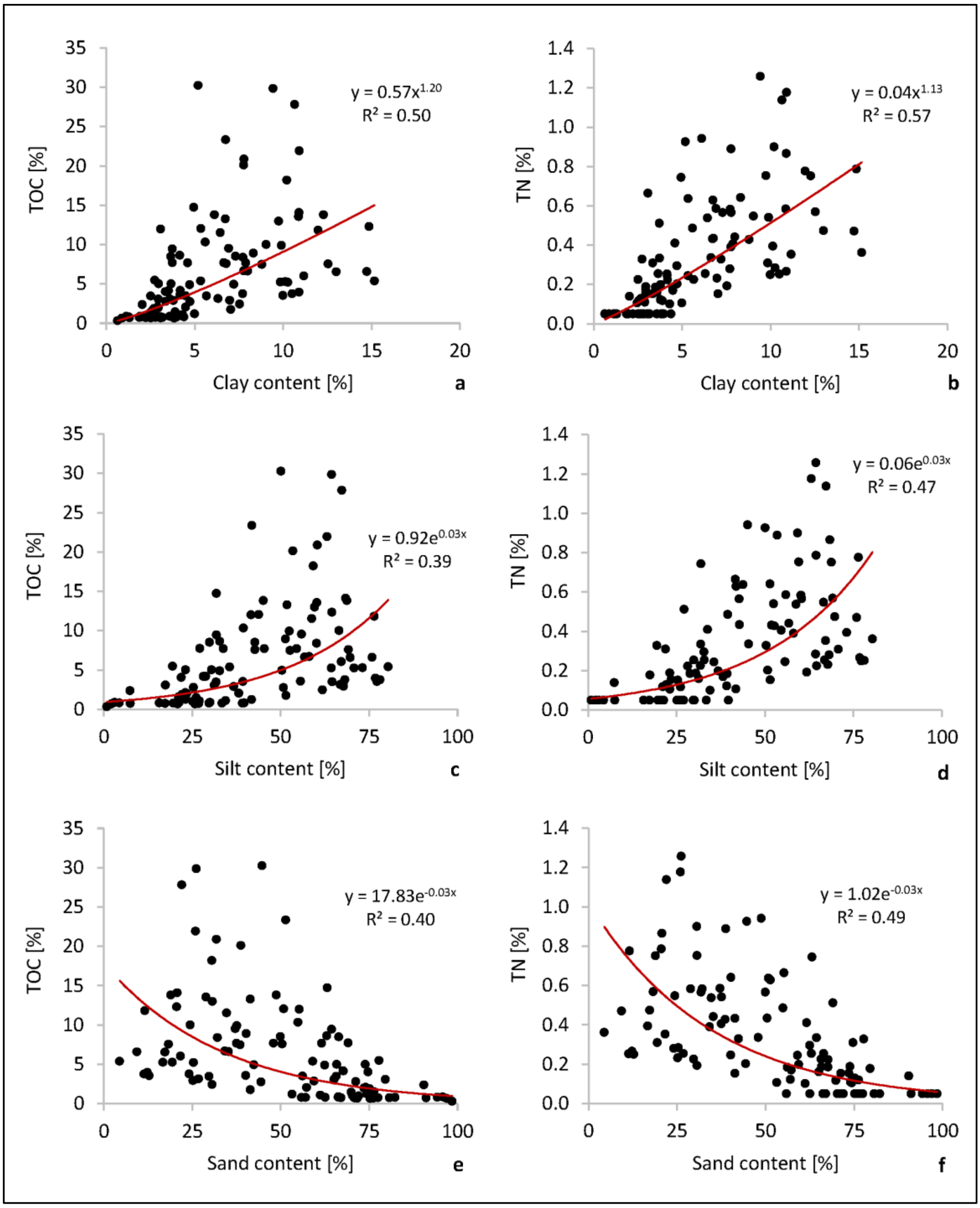

Figure S4 Grain-size distribution divided into percentages clay, silt and sand put in relation to total organic carbon (TOC) and total nitrogen (TN) contents (in percentages). Red lines indicate best-fit curves for the relationships. (**a**) TOC in relation to the clay content of the analyzed samples, (**b**) TN in relation to the clay content of an analyzed sample. Note that these two relationships (**a** and **b**) are only valid for a clay content up to 15%. (**c**) TOC content in relation to the silt content, (**d**) TN in relation to the silt content, (**e**) TOC in relation to the sand content, and (**f**) TN in relation to the sand content of an analyzed sample.

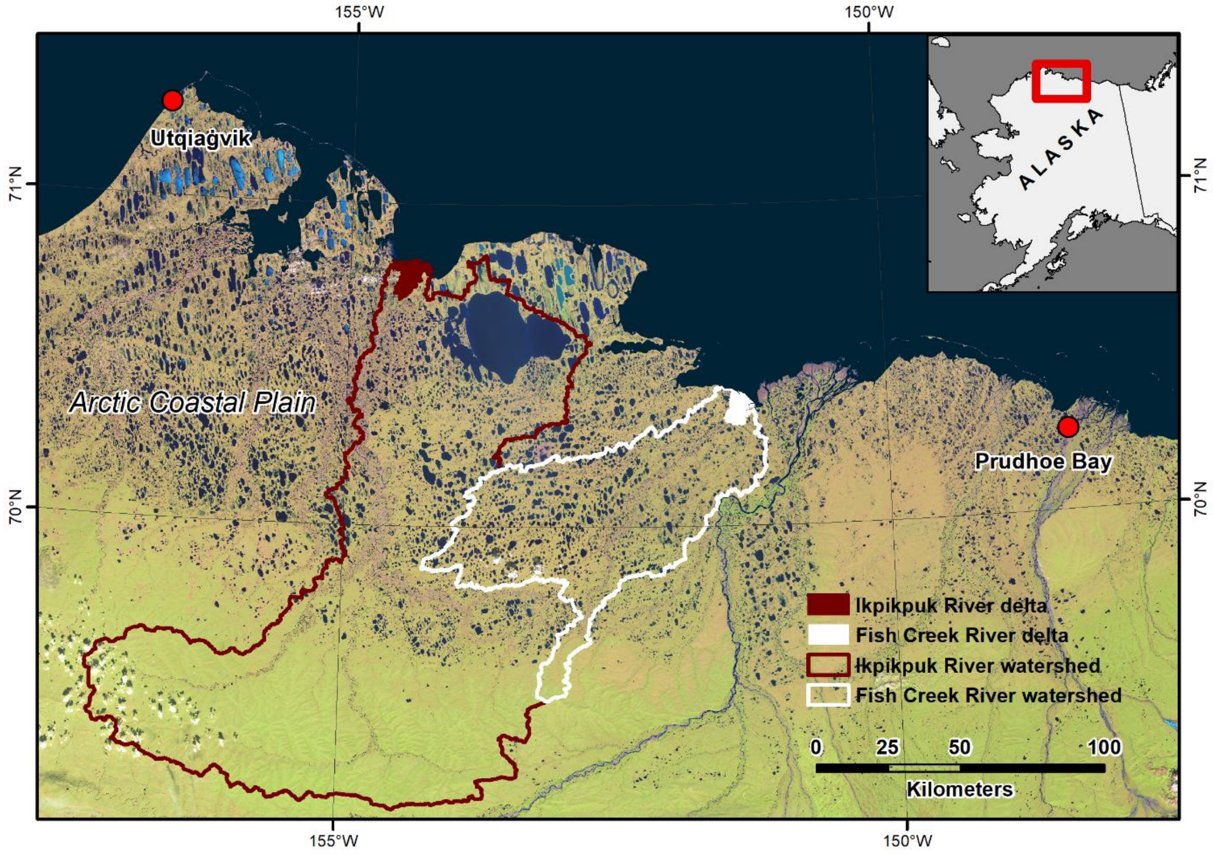

Figure S5 Watersheds of the Fish Creek (white) and Ikpikpuk River (red) on the Arctic Coastal Plain in northern Alaska on a Landsat 8 mosaic.

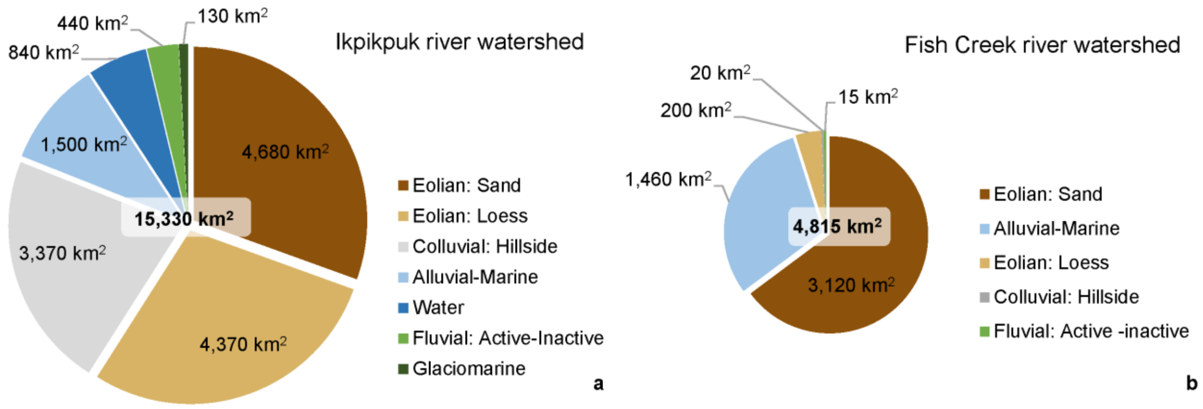

Figure S6 The surficial deposits of the (**a**) Ikpikpuk river watershed and (**b**) the Fish Creek watershed. Watershed size according to Figure S5 and surficial deposit information based on Jorgenson et al. [3].

References

1. Santoro M, Strozzi T (2012) Circumpolar digital elevation models> 55° N with links to geotiff images, ESA data user element - permafrost. Pangaea, Data Publisher for Earth and Environmental Science. doi:10.1594/PANGAEA.779748

2. Jones BM, Arp CD, Whitman MS, Nigro D, Nitze I, Beaver J, Gädeke A, Zuck C, Liljedahl A, Daanen R, Torvinen E, Fritz S, Grosse G (2017) A lake-centric geospatial database to guide research and inform management decisions in an Arctic watershed in northern Alaska experiencing climate and land-use changes. Ambio:1-18. doi:10.1007/s13280-017-0915-9

3. Jorgenson M, Yoshikawa K, Kanevskiy M, Shur Y, Romanovsky V, Marchenko S, Grosse G, Brown J, Jones B (2008) Permafrost characteristics of Alaska. In: Proceedings of the Ninth International Conference on Permafrost, 2008. University of Alaska: Fairbanks, pp 121-122

4. Carter LD (1981) A Pleistocene sand sea on the Alaskan Arctic coastal plain. Science 211 (4480):3131-3383. doi:10.1126/science.211.4480.381

5. Mann DH, Groves P, Reanier RE, Kunz ML (2010) Floodplains, permafrost, cottonwood trees, and peat: What happened the last time climate warmed suddenly in arctic Alaska? Quaternary Sci Rev 29 (27):3812-3830. doi:10.1016/j.quascirev.2010.09.002
